# Supplementary material for: Modeling glioblastoma heterogeneity as a dynamic network of cell states
Source: Mol Syst Biol. 2021 Sep 16;17(9):e10105. doi: 10.15252/msb.202010105 (PMC8444284; doi:10.15252/msb.202010105)
Supplement: Supplementary file 6 — Source Data for Figure 5 [file MSB-17-e10105-s004.zip › Figure5A_sourcedata/GSEA_3017/hallmarks_stateF.GseaPreranked.1621934628066/HALLMARK_PEROXISOME.html]

Details for gene set HALLMARK\_PEROXISOME[GSEA]

|  || Dataset | state33017 |
| Phenotype | NoPhenotypeAvailable |
| Upregulated in class | na\_neg |
| GeneSet | HALLMARK\_PEROXISOME |
| Enrichment Score (ES) | -0.2441188 |
| Normalized Enrichment Score (NES) | -0.9123036 |
| Nominal p-value | 0.5546219 |
| FDR q-value | 0.6392259 |
| FWER p-Value | 1.0 |
Table: GSEA Results Summary

  

Fig 1: Enrichment plot: HALLMARK\_PEROXISOME      
 Profile of the Running ES Score & Positions of GeneSet Members on the Rank Ordered List

  

| PROBE | GENE SYMBOL | GENE\_TITLE | RANK IN GENE LIST | RANK METRIC SCORE | RUNNING ES | CORE ENRICHMENT || 1 | GSTK1 |  |  | 312 | 0.503 | -0.0597 | No |
| 2 | SCP2 |  |  | 353 | 0.485 | -0.0007 | No |
| 3 | PRDX5 |  |  | 419 | 0.463 | 0.0435 | No |
| 4 | SLC25A4 |  |  | 836 | 0.340 | -0.0884 | No |
| 5 | ACOX1 |  |  | 912 | 0.324 | -0.0706 | No |
| 6 | CADM1 |  |  | 935 | 0.320 | -0.0298 | No |
| 7 | ATXN1 |  |  | 1138 | 0.286 | -0.0746 | No |
| 8 | ABCD3 |  |  | 1381 | 0.259 | -0.1418 | Yes |
| 9 | PEX2 |  |  | 1463 | 0.251 | -0.1383 | Yes |
| 10 | ITGB1BP1 |  |  | 1501 | -0.259 | -0.1138 | Yes |
| 11 | ERCC1 |  |  | 1530 | -0.267 | -0.0840 | Yes |
| 12 | FADS1 |  |  | 1682 | -0.312 | -0.1021 | Yes |
| 13 | CTPS1 |  |  | 1692 | -0.316 | -0.0561 | Yes |
| 14 | SMARCC1 |  |  | 1810 | -0.369 | -0.0498 | Yes |
| 15 | TOP2A |  |  | 2246 | -1.562 | 0.0031 | Yes |
Table: GSEA details [plain text format]

  

Fig 2: HALLMARK\_PEROXISOME: Random ES distribution      
 Gene set null distribution of ES for **HALLMARK\_PEROXISOME**
